# Nursing identity and patient-centredness in scholarly health services research: a computational text analysis of PubMed abstracts 1986–2013

**DOI:** 10.1186/s12913-014-0660-8

**Published:** 2015-01-22

**Authors:** Erica Bell, Steve Campbell, Lynette R Goldberg

**Affiliations:** Wicking Dementia Research and Education Centre, University of Tasmania, Private Bag 143, Hobart, Tasmania 7001 Australia; School of Health Sciences, University of Tasmania, Locked Bag 1322, Launceston, Tasmania 7250 Australia

**Keywords:** Nursing identity, Health services research, Patient-centredness, Computational linguistics

## Abstract

**Background:**

The most important and contested element of nursing identity may be the patient-centredness of nursing, though this concept is not well-treated in the nursing identity literature. More conceptually-based mapping of nursing identity constructs are needed to help nurses shape their identity. The field of computational text analytics offers new opportunities to scrutinise how growing disciplines such as health services research construct nursing identity. This paper maps the conceptual content of scholarly health services research in PubMed as it relates to the patient-centeredness of nursing.

**Methods:**

Computational text analytics software was used to analyse all health services abstracts in the database PubMed since 1986. Abstracts were treated as indicative of the content of health services research. The database PubMed was searched for all research papers using the term “service” or “services” in the abstract or keywords for the period 01/01/1986 to 30/06/2013. A total of 234,926 abstracts were obtained. Leximancer software was used in 1) mapping of 4,144,458 instances of 107 concepts; 2) analysis of 106 paired concept co-occurrences for the nursing concept; and 3) sentiment analysis of the nursing concept versus patient, family and community concepts, and clinical concepts.

**Results:**

Nursing is constructed within quality assurance or service implementation or workforce development concepts. It is relatively disconnected from patient, family or community care concepts.

**Conclusions:**

For those who agree that patient-centredness should be a part of nursing identity in practice, this study suggests that there is a need for development of health services research into both the nature of the caring construct in nursing identity and its expression in practice. More fundamentally, the study raises questions about whether health services research cultures even value the politically popular idea of nurses as patient-centred caregivers and whether they should.

## Background

### Defining identity

Identity has been a strong preoccupation of philosophy and social science enquiry for hundreds of years, both individual identity and collective or group identity. This is because identity is thought to shape individual and social behaviour [1-4]. One of the most influential theories of identity comes from the work of French philosopher Foucault on whom the Library of Congress holds over 10,000 items. Foucault defined individual and collective identity as a social construct that is created in language. In Foucault’s writings, historical and social processes work to actively create these identities, operating sometimes subliminally through particular kinds of knowledge to shape what assumptions people accept about themselves and others [5,6]. An example of this, important in this study, is how nursing and patient care have been constructed over the last 25 years in the discipline of health services research, as captured in a particular database PubMed.

In discursive psychology, which builds on the work of Foucault, identity is also understood to be about how language is used to create and position the self in relation to the world [7-9]. This second aspect of identity is also important to this study. In this study, the focus is upon how nursing identity is positioned in relation to other concepts in the discipline of health sciences as reflected in PubMed [8,9]. Nursing is seen as positioned in the language of health sciences research in ways that create particular kinds of “story-lines” that suggest professional attributes [7-9]. Of particular interest in this study is how this identity may be measured, and whether and how it offers insights into the quality agenda in healthcare in which patient-centred care is perceived as critical.

### Definitions of identity in the nursing identity literature

Identity has also been defined extensively in the nursing literature. The body of literature about nursing identity is a growing one, albeit one that sometimes lacks methods and data to support its hypotheses. For the period 1^st^ January 2008 to 5^th^ of September 2013, a total of 505 papers are listed in PubMed including the terms “nursing” OR “nurse” OR “nurses” AND “identity.” A recent review of nursing identity has made a distinction between the professional self-identity of nurses and the public identity or image of nursing [10]. The public image has been described as partly created by default by nurses themselves who have not always participated in public discourse. The professional self-identity of nurses is a complex construct that includes, but is not limited to, this public image, work contexts, work values, education and social or cultural values [10]. Specialist nurses such as mental health nurses have been found to have an identity comprised of a cluster of “identity characteristics” [11]. The acquisition of a nursing professional identity through processes of “professional socialisation” is known to be complex and multi-faceted and is the focus of a substantial part of the nursing identity literature [12]. Nursing identity has also been described as a measureable self-concept that can be “manipulated” to help improve nursing retention, although sound psychometrically validated instruments are needed [13,14].

### The importance of nursing identity to the quality of healthcare

Nursing identity is seen as having a fundamental importance for the quality of healthcare. Nursing identity has been strongly linked to student retention in nursing programs [15]. Recruitment and retention problems in, for example, mental health nursing have been linked to the ambiguity of the mental health nursing role [16]. Nursing identity has also been constructed as important to whether and how nurses expand their leadership roles and skills, with positive implications for patient care [17,18]. Ideas about professional identity and roles shape nursing service management in, for example, the contested area of optimum skill mix and nursing staffing levels for patient outcomes [19]. Nursing identity has also been seen as important to growing collaborative research involving academic and practice settings, which in turn are perceived as helping to improve the quality of patient care [20]. Nursing identity has even been shown to shape the extent to which nurses support healthcare reform affecting their roles [21].

### Patient-centred care and its importance to nursing identity

One of the most important constructs in the popular and scholarly literature about nursing identity is “patient-centred care.” Yet it is also one of the most contested constructs. A recent review found that nurses, physicians and managers all used the discourse of “patient-centred care” to suggest that their own professional group was patient-centred while other professional groups were not [22]. In the language of policy-makers, “patient-centredness” may be a lever for achieving change in professional attitudes, as in the United Kingdom’s public debate about graduate nurses being “too posh to wash” or deliver essential nursing care [23,24]. In such a politicised debate in the media, problems with nursing recruitment, as well as nursing care, have been seen as being caused by the increasingly academic nature of nurse education and the specialisation of the nursing professions [25]. Yet in the nursing literature, the role of “essentialist caring values” is often described as important to the future of nursing [26].

However, patient care as a component of professional identity—the degree to which caring is seen as a part of nursing professional identity—has had relatively less attention in the contemporary nursing identity literature. Caring in nursing is articulated as being linked to task-oriented approaches to nursing and being grounded in the relational empathy and connection between the professional nurse and the patient [27]. A “dimensional analysis” of patient-centred care describes it as including an eclectic range of elements, from providing a comforting room design to emotional support to attention to individualised meals to supports for patient decision-making [28]. The authors of this analysis considered 69 papers published between 2000–2006 and found that the common themes of patient-centred care involved complex and sustained attention in nurse-patient interactions to alleviating vulnerabilities, as well as therapeutic engagement based on a knowledge of, and relationship with, the patient [28]. Thus, in the nursing literature, “patient-centred” care has a strongly individual patient-nurse meaning.

Beyond the literature on nursing identity as such, nursing theory and knowledge development have traditionally included a strong discourse of “caring science scholarship” [29]. The construct of caring is a multi-faceted one that has been the subject of different conceptualisations and theoretical frameworks since the 1990s at least [30-33]. Watson has described the dominant construct of caring as involving emotional and physical labour components, or expressive and instrumental elements, and argued that such intuitive constructs can be operationalized and measured [34,35]. The construct has been most notably applied using instruments such as the Caring Dimensions Inventory to measure student perceptions of caring as having both psycho-social and professional/technical dimensions [34-37]. By 2001, 16 such instruments were reviewed in a monograph on assessing and measuring caring [38]. Yet there is a perceived divide between qualitative and quantitative approaches to researching caring [35] and caring research has been criticised for failing to yield to scientific advances in knowledge [39].

### Perceived threats to the patient-centredness of care and the quality of healthcare

Despite the importance of “patient-centred” care to nursing identity, it is clear that it is perceived as being under threat in ways that threaten the quality of healthcare. For example, in hospital settings, studies have suggested that only around a third of the time of different nursing professionals is spent on direct patient care, despite the growing complexity of these direct care needs: admission and assessment, hygiene and patient/family interaction, medication and IV administration and procedures [40]. Concerns about the deregulation of direct patient care in, for example, areas of growing care complexity such as aged care, position a declining valuing of direct patient care and care workers as part of the problem of care quality [41]. The whole evidence-based practice movement has been seen as devaluing the complex interpersonal element of the caring components of nursing [42]. That is, the emphasis on evidence-based practice, outcomes-based practice, and quantifiable efficiencies has been seen as ultimately threatening the fundamental “caring” strengths of nursing identity and practice, and the quality and safety agenda [43]. Industrially, “caring” in nursing identity is also seen as under threat by new regimes of economic management, characterised by widespread cuts to services and staff reductions [44].

This is not to argue that patient-centredness has an unproblematic relationship to patient outcomes. The evidence is mixed regarding the efficacy of patient-centredness as a style of doctor-patient communication [45]. The asymmetry between doctor and patient is thought to have deep and even possibly justified roots in the clinical authority of the doctor [45]. However, as the foregoing discussion demonstrates, the nursing literature has defined patient-centredness far more broadly: in terms of tasks of caring and verbal and non-verbal interaction. “Patient-centeredness” has deep roots in the whole identity of the nursing profession itself in which a nurse has a pastoral authority for broader patient health and well-being. In a context in which it is known that health outcomes are shaped by wider determinants of health, it seems likely that 1) a narrow definition of patient-centredness will not achieve real changes in patient outcomes, and 2) the patient outcomes of a broader definition of patient-centredness consistent with nursing caring scholarship will continue to be difficult to measure.

Notwithstanding, it is certain that ideas about nursing identity and patient-centred care will continue to shape public debate and healthcare quality agendas. For example, both the UK’s Essence of Care [46] and Australia’s Essentials of Care [47] emphasise a re-establishment of the traditional direct care roles of nurses. Underlying such debates is an uneasiness about the relevance of new nursing identities, in a context in which a disconnect between the disciplinary mission of nursing and its constituent patient communities is known to lead to the failure of health programmes [48].

### The importance of health services research to constructing nursing identity in the 21stC

The current study explores the construction of nursing identity from another perspective—that of research evidence, specifically health services research. The discipline of health services research is a particularly important field in which to examine nursing identity because it delivers the evidence that potentially shapes practice in health services. Health sciences research will increasingly be under pressure to deliver service benefits, through the emphasis on translational research of such United States-based agencies as the Institute of Medicine, Agency for Healthcare Research and Quality, and National Institutes of Health. The entire translational research movement, originally about better articulation of research benefits from the science laboratory “bench” to clinical practice “bedside” [49,50] has developed over the last quarter of a century into new sub-disciplines of health research ensuring that health sciences research also includes health services research [51].

Accordingly, this study aims to explore to what extent the emerging discipline of health services research has positioned nurses as patient caregivers over the last quarter of a century in which over 200,000 papers in this discipline have been published. As such, this paper responds to calls in a recent review of nursing identity for more conceptually-based mapping of nursing identity constructs, capturing the subtlety of changes over time [13]. It also responds to the need for more information for nurses that can help them reflect on, and shape, the construction of their own identity, including patient-centredness, particularly given its importance in healthcare reform policy, nursing education and training, and care efficacy [10,52].

### Machine-driven text analytics

The size of the corpus of abstracts in PubMed relevant to health services is prohibitive for traditional manual methods of qualitative analysis. However, automated “natural language processing” or “computational linguistics” — the methods that inform the software used in this study — has developed over the last 30 years to offer ways of handling such unstructured datasets [53]. Natural language processing can be described as a diverse range of automated computational techniques for analysing and representing language. It underpins technological innovations such as Google and IBM’s Watson, as well as Apple’s Siri [54]. This section does not reproduce the content of reviews of the field published elsewhere [54], instead focussing on literature offering examples of applications in the health sciences and the justification for our choice of Leximancer software.

As of 15 April there were only 1,034 journal papers listed in the database PubMed with the terms “computational linguistics,” “natural language processing,” or “text analytics” in the title or abstract, 655 of which were published since 2008. These suggest that, in health, applications have been diverse and include: 1) exploration of theories in the light of social media [55]; 2) analyses of clinical texts for the purposes of classification [56], symptom description [57] and diagnosis [58], the analysis and prediction of patient outcomes [59,60] and evaluation of the extent of utilisation of evidence in health practice [61]; 3) the analysis of healthcare experiences and behaviours using popular media such as YouTube [62]; and 4) clinical decision support [63]. This study takes a socio-cultural perspective, focussing on the extent to which patient-centred nursing is under-represented in the discipline of health services as represented in the PubMed database and as such may be described as a form of “evidence surveillance.” In this study, software associated with natural language processing is used to explore a theoretical construct — nursing professional identity as it relates to patient-centredness in the PubMed corpus.

It is acknowledged that those wanting to learn how to do natural language processing (NLP) can access the open source Natural Language Toolkit (version 2.0 http://www.nltk.org) using Python programming language (version 2.7, http://www.python.org) [64]. However, not all researchers will be able to become proficient computer programmers or have the resources to access computer programmers. Some researchers may also choose to use high quality standardised software products developed by advanced programmers, particularly for complex procedures such as concept mapping and sentiment analysis, to help ensure standardised results that are supported by detailed manuals and (ideally) technical validity studies. For these researchers we suggest consideration of “text analytics” software available on the market, designed using the methods of NLP. A summary of these “text analytics” products is available by market analysts [64] and there are also digital libraries available at http://dirtdirectory.org/.

The software package Leximancer [65], originally created at the University of Queensland in Australia, includes two key functions—concept mapping and sentiment analysis—that are fairly standard in the field. Leximancer compares favourably with other products listed in the Hurwitz report [64] when criteria such as scholarly literature modelling its application, cost, installation and technical running problems and support, as well as user friendliness, are considered. We have tried and tested other products including the costly IBM SPSS Modeller Premium software (Version 15.0, http://www.spss.com.hk/software/modeler/). We have found that other products do not have the scholarly validity study [66] or body of published scholarly applications supporting Leximancer’s concept analysis and sentiments analysis procedures [67-74]. However, there has not yet been an application of Leximancer to the corpus of health service research abstracts, although the software offers a novel technique for mapping this large language dataset. Notwithstanding, the software suffers from the limitations of other NLP approaches: a lack of “real world” and common-sense knowledge about the contextualised meanings of words.

Leximancer is Bayesian-based software that “learns” from an uploaded dataset that it reads iteratively. For concept mapping, Leximancer creates a network of concepts defined in “text blocks” of about a paragraph in size, normally three sentences, but defined by a process of machine learning (described below). In Leximancer, a text block is the unit of analysis. An abstract may contain one or more text blocks and one text block may contain one or more concepts, just as a sentence can contain more than one concept. In sentiment analysis, Leximancer maps the frequency and co-occurrence of concepts with an in-built thesaurus of sentiment terms (negative versus positive). In more technical terms, Leximancer is described as an unsupervised approach to transforming lexical co-occurrence information from language data into semantic patterns. There are two kinds of co-occurrence information extraction in Leximancer’s automated procedures—semantic and relational—each with a different algorithm [66]. These algorithms employ nonlinear dynamics and machine learning as summarised in the following technical overview:

A unified body of text is examined to select a ranked list of important lexical terms on the basis of word frequency and co-occurrence usage. These terms then seed a bootstrapping thesaurus builder, which learns a set of classifiers from the text by iteratively extending the seed word definitions. The resulting weighted term classifiers are then referred to as concepts. Next, the text is classified using these concepts at a high resolution, which is normally every three sentences. This produces a concept index for the text and a concept co-occurrence matrix. By calculating the relative co-occurrence frequencies of the concepts, an asymmetric co-occurrence matrix is obtained. This matrix is used to produce a two-dimensional concept map via a novel emergent clustering algorithm. The connectedness of each concept in this semantic network is employed to generate a third hierarchical dimension, which displays the more general parent concepts at the higher levels [66].

This means that, for example, for the concept of nursing, Leximancer works by merging similar terms e.g. nurses, nursing, nursing assistant. The classifications of nursing will be developed from the text: if “physician’s assistant” is used synonymously with “nurse” the term may be learned by Leximancer and integrated into a nursing concept. The analyst also scrutinises the output at different stages to identify the concept seed words and the thesaurus terms Leximancer produces. Terms that are obviously the same can be merged manually. This demonstrates an important feature of Leximancer: it is automated but also features multiple interactive data viewing windows that allow analyst scrutiny and intervention.

The current study is therefore not a study of types of research but rather of concepts in the corpus of abstracts about health services in PubMed. The advantage of a concept analysis is precisely that it does not involve a valuing or critiquing of research using familiar typologies of research such as quantitative or qualitative research or any other way of describing research methods or research disciplines. Rather, this study measures the prevalence of particular concepts that cut across these different types of research. An example of a text block classified by Leximancer under the nursing concept, and tending to emphasise nursing service structures rather than the immediate pastoral dimensions of caring in nursing, is as follows:

PMID: 3228766

409. Can J Nurs Adm. 1988 Oct;1(3):16–8.

Ambulatory care nursing. A new approach.

McMaster DC, Greer PM, Beanlands HE.

This article describes one hospital’s experience with integrating inpatient and outpatient nursing services. Nursing services integration enabled nursing management to combine the nursing resources allocated to the inpatient and outpatient components of a clinical service under the direction of one nurse manager. This new and creative approach was implemented in thirteen clinical services at the Victoria General Hospital, Halifax. This organizational structure was considered to be an effective approach for managing ambulatory care nursing services. The introduction of integration facilitated a change in the ambulatory care nursing role. It also provided for increased continuity of patient care and afforded nurses the opportunity to practice in another setting. Nursing services integration is considered a more effective approach for managing nursing resources.

Further technical details about Leximancer analysis are available in the validity study [66].

## Methods

In a sentence, concept mapping in Leximancer is about understanding semantic relationships within the entire network of concepts that exist in a language dataset. Sentiment analysis is about understanding the extent to which a concept is semantically proximate to sentiment words generally, as well as the extent to which a concept is semantically proximate to positive versus negative words. In terms of our theoretical framework, we agree that the definition of a concept used in concept mapping is itself the result of epistemologies [75].

Concept mapping and content analysis have traditionally been defined by theoretical frameworks, such as those of Saussure [76], which assume human, manual methods of textual analysis. However, while Leximancer is a machine-based method it also has certain embedded epistemological assumptions, namely that language is an artifact of human behaviour that can be measured as a semantic network, albeit only as a starting point for qualified interpretation and further data mining. We do not believe that the conflict in concept mapping approaches between the analytic school and the postmodern deconstructionist one [75] — a conflict essentially between the study of formal structuralist approaches to language such as linguistics, versus the deconstruction of texts associated with philosophers such as Derrida [77] — is at all useful. Rather, we believe that machine-based approaches based on the field of natural language processing, as used in this study, need to be framed by theoretical constructs — in this study from the caring and identity literature — and include a section on the limitations of machine-based findings.

### Research questions

The research questions were ‘How is content about nursing, especially as it relates to patient-centredness, related to other content in health services research abstracts in PUBMED?’ and ‘What are the implications for understanding whether patient-centred nursing is under-represented in the health services literature?’

### Data sources

The study was limited to how health services research is constructed in PubMed, an authoritative database of 22 million citations, including from MEDLINE, maintained by the National Center for Biotechnology Information at the United States’ National Library of Medicine, based at the National Institutes of Health. As such, the study was designed to be an analysis of how health services research constructs nursing identity and patient-centredness in a particular database — PubMed — not an exhaustive study of all scholarly health services literature.

The method involved treating PubMed abstracts as indicative evidence of the content of health services research in this database. The database PubMed was searched for all research papers using the term “service” or “services” in the abstract or keywords for the period 01/01/1986 to 30/06/2013. The downloaded abstracts were added to a SQLite database used to generate CSV files with abstracts batched in three-year periods (except for 2013 which was batched from 1/1/13-31/6/13). Manual checks of data consistency were conducted that also removed duplicate abstracts. This involved an iterative process in which automated searches of duplicates were complemented by manual spot checks and took an estimated six weeks of full-time work. A total of 234,926 abstracts were obtained. Table 1 provides the counts of abstracts for each period of the analysis. The period 1986–1988 was selected as the first period of the study to allow the study to cover a span of at least a quarter of a century.

**Table 1:** **Counts of abstracts, by year**

**Jan-June 2013**	**8983**
2010-2012	50638
2007-2009	39359
2004-2006	32298
2001-2003	25357
1998-2000	22641
1995-1997	19342
1992-1994	15976
1989-1991	12887
1986-1988	7445
Total	234926

### Analytical procedure

The analysis proceeded using three standardised stages that involved quantification of the conceptual content of the abstracts using Leximancer software [65].

#### Stage 1: concept mapping

This stage obtained a conceptual overview of the health service abstracts. Leximancer was used to obtain frequency and co-occurrence statistics for all concepts in the abstracts, as well as visualisations of these data in a concept map. This involved some “concept cleaning” by the authors in which only irrelevant words that related to the common structural features of abstracts such as “conclusions” and “method” were discarded as mapping concepts. Country names such as USA were not included as mapping concepts as country-based themes were beyond the scope of the study. The concept map was designed to show what concepts were found, and their proximity to one another, in the larger dataset of 234,926 abstracts for health services research. This stage mapped a total of 4,144,458 instances of 107 concepts in 1,311,805 text blocks of about a paragraph in size. It also suggested typical connections (or common storylines) between the nursing concept obtained and multiple other concepts in the health services abstracts.

#### Stage 2: nursing concept analysis

In this stage, the 54,507 instances of the nursing concept identified in Stage 1 were analysed for immediate associations with all other concepts in the dataset. A total of 106 types of paired co-occurrences for the nursing concept were extracted and analysed. Supporting counts and likelihood of paired co-occurrences were obtained, as well as likelihoods for the nursing concept occurring in the different sub-periods of the analysis.

#### Stage 3: sentiment analysis

This stage mapped how sentiment about nursing compared with sentiment about patient, family and community concepts as well as with more clinical concepts in the health service literature. Frequency and extent of sentiment associations with the nursing concept were compared with the sentiment associations of nine other selected concepts: clinical, community, family, hospital, medical, patients, prevention, social, treatment. The inbuilt thesaurus of positive and negative sentiment words in Leximancer was used to analyse changes over time in negative and positive sentiment associated with these 10 selected concepts. A visual representation of sentiment associations with nursing versus sentiment associations with selected other concepts, by sub-period, was obtained using a quadrant.

The process of producing the final concept map is stochastic, so the stability of findings was tested by running the data three times, consistent with the account of stability issues provided in the technical validity study [66]. Manual checks were also performed of logs of text blocks informing the software output reported here. The multiple data viewing windows available in Leximancer also facilitated iterative manual checks on the findings. For example, for each concept, the analyst can view all text blocks in isolation and also in context in the original datafile, as well as log and further analyse those individual concepts.

## Results

### Concept map

**Figure 1:**
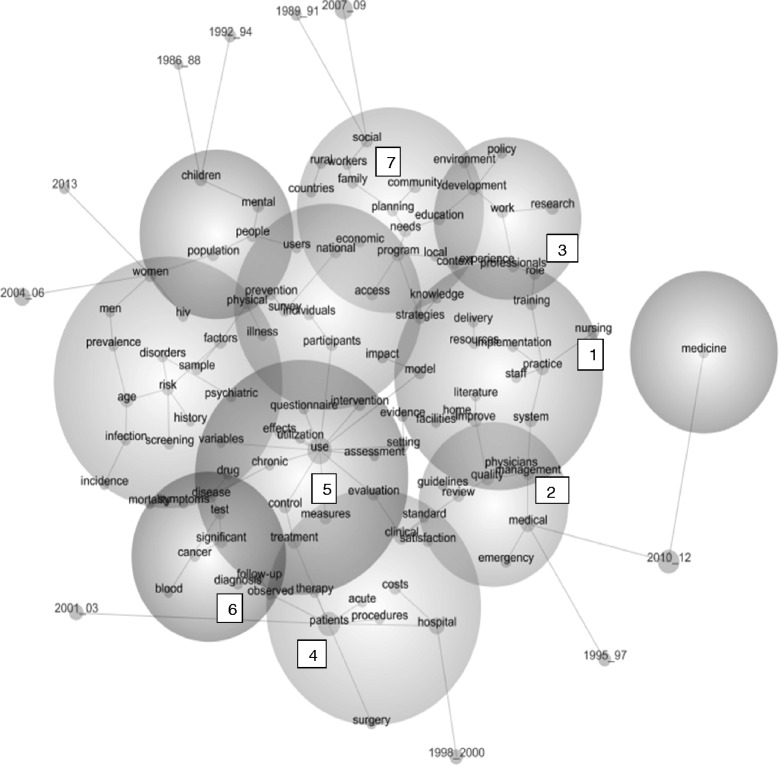
**Concept map of all 107 concepts in health services abstracts 1/1/1986-31/6/2013.** The nursing concept is identified in sphere 1, with connecting pathways to other concepts in spheres 2 and 3. The concept of patient is identified in sphere 4, with connections to concepts in spheres 5 and 6. The concept of family is in sphere 7.

Figure 1 maps all 4,144,458 instances of the 107 concepts found in 1,311,805 text blocks of about a paragraph in size. The concept map should be considered as a spatial representation of the semantic proximity of these 107 concepts. The location of a concept and its time period is determined by its placement relative to all other concepts [65]. The grey lines suggest the more typical semantic pathways or “storylines” involving multiple concepts (not necessarily the most frequent pairs of concepts). The size of the spheres is designed to accommodate the spatial semantic placement of the concepts, i.e. the size of the spheres denotes these boundaries only. The size of a grey dot indicates the extent to which that concept co-occurs with all other concepts. This overall co-occurrence is different from the paired co-occurrences examined later which are about immediate relationships between two concepts — not the relatedness of one concept to all other concepts in a dataset. The map is designed to offer a bird’s eye view of the data, meant to be supplemented by the supporting data in the tables.

Figure 1 suggests that the nursing concept (in sphere 1) is typically connected to these storylines:

Nursing → practice and, through “practice,” branching out to multiple other storylines (like the spokes of a wheel around the practice concept), such as system → management (moving into sphere 2) → quality OR staff OR implementation (in sphere 1) → resources → delivery OR training → role (moving into sphere 3) → professionals.

This suggests that nursing has tended to be constructed by the health service research within quality assurance or service implementation or workforce development concepts. It suggests that service research does not tend to construct nursing in terms of broader patient, family or community care storylines.

The location of the patient concept in sphere 4 suggests health service research constructs patients within the following typical storylines to do with biomedical interventionist, cost utility and research measurement storylines (again, moving round the concept of patient like the spokes of a wheel):

Patients → therapy → treatment (moving into sphere 5) → control → use

Patients → acute (within sphere 4)

Patients → procedures → hospital → costs (within sphere 4)

Patients → surgery (within sphere 4)

Patients → observed → diagnosis (moving into sphere 6)

The concept map in Figure 1 suggests also that family and community concepts, like the patient concept, are relatively less well connected to the nursing concept. Both the family and community concepts (in sphere 7) are linked (in separate spokes) only to the planning → needs → program concepts. This suggests that when these concepts occur they typically do so within quite singular planning storylines.

Table 2 gives counts of instances of all of the 107 concepts (bearing in mind a text block can contain more than one instance of a concept) and relevance of that concept. “Relevance” is the percentage of text blocks in which that concept appears, relative to the most frequent concept entity on the map, i.e., relevance is the count of a concept divided by the single highest count value which always therefore has a relevance of 100%. Therefore it is the relativities between the concepts that are of interest. Table 2 suggests that the storylines about nursing within the health service abstracts need to be understood in the context of how service research constructs itself. The most common concepts in the service research are intervention use (which includes words to do with utility or usefulness), clinical response and clinical setting concepts (such as “hospital,” “medical,” and “clinical”) — concepts with 69% to 29% relevance. “Patients” is the second most relatively common concept in Table 2, notwithstanding its distance from the nursing concept in Figure 1. Table 2 also shows the community concept is actually one per cent more common than nursing, whatever its proximity to the nursing concept in Figure 1.

**Table 2:** **Counts and relevance of instances of concepts**

**Concept**	**Count**	**Relevance**	**Concept**	**Count**	**Relevance**
use	217339	69%	cancer	31290	10%
patients	194810	62%	surgery	30991	10%
hospital	110504	35%	implementation	30803	10%
medical	91944	29%	individuals	30377	10%
clinical	91823	29%	drug	29179	9%
treatment	73690	23%	resources	28540	9%
research	71312	23%	professionals	28134	9%
children	67849	22%	home	28112	9%
practice	65274	21%	diagnosis	28008	9%
program	62746	20%	hiv	26293	8%
community	57096	18%	disorders	26159	8%
system	56844	18%	countries	25570	8%
age	56635	18%	assessment	25055	8%
improve	56078	18%	strategies	25049	8%
people	55593	18%	prevalence	24919	8%
significant	55159	17%	screening	24412	8%
women	54752	17%	delivery	23784	8%
nursing	54507	17%	staff	22767	7%
quality	54136	17%	therapy	22618	7%
population	53829	17%	evidence	22468	7%
disease	51932	16%	mortality	22115	7%
factors	51551	16%	knowledge	22008	7%
costs	51029	16%	utilization	21967	7%
medicine	50018	16%	physicians	21909	7%
needs	49194	16%	standard	21772	7%
mental	49072	16%	questionnaire	21710	7%
measures	48653	15%	infection	21618	7%
risk	48644	15%	rural	20922	7%
control	47938	15%	effects	20750	7%
national	47507	15%	symptoms	18978	6%
social	46583	15%	psychiatric	18620	6%
management	46441	15%	physical	18266	6%
education	46276	15%	users	17903	6%
work	45608	14%	local	17609	6%
survey	44941	14%	observed	17356	6%
intervention	44208	14%	environment	17297	5%
participants	43618	14%	facilities	17295	5%
model	42407	13%	procedures	17166	5%
review	41351	13%	acute	16859	5%
access	40580	13%	workers	16638	5%
prevention	40396	13%	chronic	16031	5%
development	39687	13%	satisfaction	16010	5%
test	39575	13%	men	15957	5%
evaluation	36279	12%	literature	15520	5%
planning	36084	11%	follow-up	15510	5%
emergency	35873	11%	illness	15173	5%
family	35779	11%	economic	15141	5%
setting	35535	11%	incidence	14530	5%
policy	34369	11%	blood	14010	4%
experience	33156	11%	variables	13996	4%
training	31694	10%	guidelines	13238	4%
sample	31611	10%	history	12111	4%
impact	31578	10%	context	11561	4%
role	31297	10%	**Total**	4,144,458

**Table 3:** **Counts and likelihood of the nursing concept, by study sub-period**

**Sub-period**	**Count**	**Likelihood**
1995-1997	4669	5%
2001-2003	6542	5%
1998-2000	5462	5%
2004-2006	8264	5%
1992-1994	3419	4%
1989-1991	2449	4%
2007-2009	9373	4%
2010-2012	11283	4%
2013	1991	3%
1986-1988	1055	3%
**Total**	54,507	

### The nursing concept

Table 3 provides the counts of all 54,507 instances of the nursing concept for each sub-period of the analysis from 1986 to June 2013. The likelihood percentage gives the percentage of all text blocks in a sub-period that contain the nursing concept. It suggests that, regardless of increases in the amount of health services research suggested by Table 1, the proportional share of all research that is about nursing is actually a little lower in the last five years (2007-June 2013) than in the period just before (2001–2006) or indeed even from 1995–2000. That is, if Figure 1 and Table 2 suggest nursing has not been central to patient, family or community care storylines in the health services research, Table 4 suggests that the nursing concept itself has not been increasing in the growing field of health service literature.

**Table 4:** **Counts and likelihood of paired co-occurrences for the nursing concept**

**Nursing concept pair**	**Count**	**Likelihood**	**Nursing concept pair**	**Count**	**Likelihood**
home	7045	25%	guidelines	609	5%
staff	3734	16%	medical	4202	5%
role	4711	15%	evidence	1017	5%
professionals	3612	13%	individuals	1340	4%
practice	7286	11%	use	9567	4%
work	4878	11%	sample	1385	4%
education	4891	11%	illness	656	4%
facilities	1695	10%	national	2027	4%
knowledge	2125	10%	system	2410	4%
satisfaction	1507	9%	chronic	654	4%
training	2941	9%	observed	703	4%
physicians	2000	9%	users	714	4%
literature	1288	8%	factors	2023	4%
experience	2621	8%	follow-up	606	4%
questionnaire	1710	8%	access	1580	4%
community	4458	8%	effects	804	4%
setting	2769	8%	significant	2131	4%
workers	1289	8%	cancer	1176	4%
acute	1247	7%	measures	1812	4%
development	2712	7%	costs	1895	4%
needs	3358	7%	procedures	631	4%
implementation	2070	7%	variables	514	4%
context	767	7%	control	1649	3%
psychiatric	1212	7%	therapy	750	3%
management	2996	6%	utilization	701	3%
delivery	1528	6%	children	2141	3%
assessment	1576	6%	history	380	3%
quality	3388	6%	countries	790	3%
clinical	5578	6%	prevention	1213	3%
environment	1048	6%	population	1564	3%
research	4271	6%	surgery	874	3%
participants	2592	6%	risk	1322	3%
model	2511	6%	age	1524	3%
hospital	6481	6%	economic	406	3%
resources	1667	6%	screening	647	3%
strategies	1447	6%	test	1036	3%
program	3489	6%	treatment	1906	3%
survey	2494	6%	symptoms	489	3%
mental	2720	6%	disease	1268	2%
social	2568	6%	women	1336	2%
family	1972	6%	diagnosis	661	2%
people	3039	5%	medicine	1179	2%
intervention	2379	5%	drug	664	2%
improve	2974	5%	infection	479	2%
rural	1073	5%	blood	267	2%
review	2118	5%	mortality	412	2%
planning	1848	5%	hiv	470	2%
patients	9888	5%	prevalence	441	2%
evaluation	1835	5%	incidence	251	2%
physical	897	5%	disorders	415	2%
impact	1549	5%	men	249	2%
standard	1064	5%	**Total**	217002	
emergency	1735	5%			
policy	1598	5%			
local	813	5%			

Table 4 gives the counts and likelihood of occurrence of all 106 concepts paired with the nursing concept in the health service abstracts (in contrast to the dominant storylines across multiple concepts suggested by Figure 1). The likelihood percentage gives the percentage of all text blocks in a pair that contain the nursing concept. For example, given we have selected the nursing concept, the related concept list includes a concept called “satisfaction” that occurs 1,507 times and has a likelihood score of 9%. The likelihood percentage of 9% means that 9% of all the text blocks with the “satisfaction” concept also contain the nursing concept. This statistic is designed to complement the count statistics so both directions of conditional probability are given [78]. Of course, given that we are dealing with the likelihood that any one paired concept will also contain the nursing concept, the likelihood percentages of all these 106 concepts will not add up to 100%.

Table 4 therefore suggests that aged care homes is most commonly paired with nursing. Manual checks were conducted to ensure that the “home” concept was overwhelmingly about aged care homes not home nursing. The next two most common nursing paired co-occurrences are with nursing staffing and nursing role concepts. Table 4 suggests that, when the patient concept occurs, it is paired with the nursing concept only 5% of the time in the service abstracts. When the community concept occurs, it is paired with the nursing concept only 8% of the time. The family concept is paired with the nursing concept only 6% of the time. In contrast, the least common paired co-occurrences for the nursing concepts — those listed as occurring in 2% of the text blocks where the paired concept occurs, are more clinical disease and diagnostic concepts, as well as more technical epidemiological research concepts such as “prevalence” and “incidence” concepts. Accordingly, Table 4 suggests that closer inspection of the nursing concept confirms not only that nursing staffing and nursing role concepts predominate, far more than patient, family and community concepts. It also suggests that the nursing concept is marginal to more clinical disease and diagnostic concepts, as well as more technical epidemiological research concepts.

### Sentiment analysis

**Figure 2:**
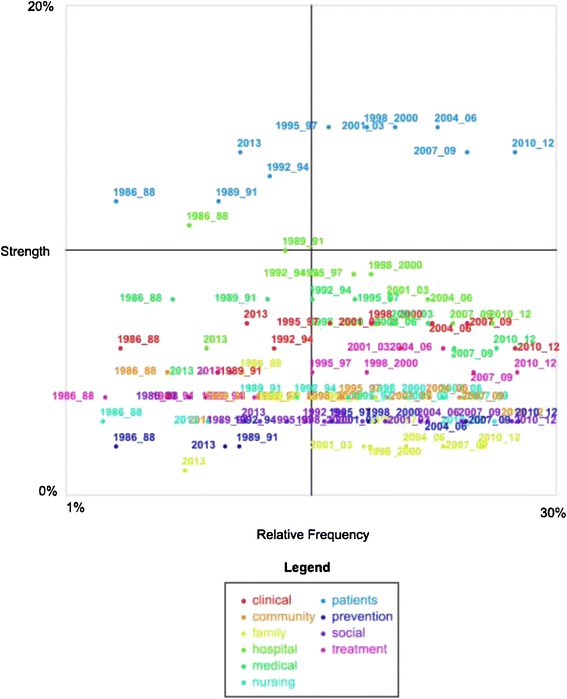
**Sentiment analysis of selected concepts, by sub-period 1/1/1986 – 31/6/2013.**

In this study, sentiment analysis is used as an adjunct or supplementary analysis only. Figure 2 is a quadrant: the x axis is a measure of the extent of sentiment association (of any kind) and the y axis is a measure of the proportion of that association that is positive (versus negative since we are dealing with only sentiment terms). The definition of the axes, adapted from the software output, is:

*Relative Frequency*: a measure of the conditional probability of the concept in a sub-period, given the category (in this case positive/negative sentiment), e.g. given we are looking at occurrences of positive or negative sentiment, how likely is it that the concept “patient” in the sub-period 1992–94 is mentioned?

*Strength*: a measure of the conditional probability of the category (in this case positive/negative sentiment) given the particular concept in a subperiod, e.g. given we are looking at occurrences of the concept “patient” in 1992–94, how often is it mentioned in positive versus negative sentiment, i.e. the 'strength' of the positive association [65]?

Understanding the central lines involves understanding the axes. The central lines that cross the graph simply suggest the middle lines of the quadrant. That is, for the x axis scale of 1% to 30%, the midpoint is the point at which 15% of occurrences of positive or negative sentiment contain the concept “patient” in a sub-period. Given the y axis is from 0-20%, the midpoint on the y axis is 10% or the point at which 10% of the content of a concept in a sub-period has positive associations (versus negative associations). Concepts in sub-periods located in the top right hand corner are therefore found more frequently with sentiment terms and are relatively more positive (which does not mean they are positive; that depends on the y scale). Concepts located in the bottom left hand corner are found less frequently with sentiment terms and are relatively more negative. Concepts that have insufficient sentiment associations of any kind do not, of course, appear.

Accordingly, the quadrant in Figure 2 visualises associations between a selection of 10 concepts: clinical, community, family, hospital, medical, nursing, patients, prevention, social, and treatment. The colour of the sub-period suggests concepts in that sub-period as indicated by the legend. The x or horizontal axis plots the relative frequency of sentiment associations of any kind, i.e., whether negative or positive, with a concept in a sub-period. The y or vertical axis plots the extent to which a given sub-period has positive associations, i.e., given a concept in a sub-period can often be found many times with sentiment associations (positive or negative), the proportion of those instances that are positive. The highest frequency for any of the selected concepts in a sub-period being found in text blocks with sentiment terms (negative or positive) is less than 30% (x axis). The proportion of positive to negative sentiment is less than 20% for all concepts in a sub-period (y axis).

Therefore, the y axis in Figure 2 suggests that “patients” is the most positive concept, followed by “hospital,” “medical,” “clinical,” and “treatment.” The other concepts are not so distinguishable in terms of proportion of positive sentiment; however, “family” tends to be the more negative concept, particularly since 1998. Accordingly, the quadrant suggests that in the discipline of health services research, as defined in PubMed abstracts, biomedical approaches, rather than community and family approaches have attracted greater proportions of positive sentiment. This is not the same as saying any of these concepts are generally positive: the midpoint of this scale of 10% means that only 10% of the sentiment associations are positive versus negative (i.e., 90% are negative). The generally negative language in research should be viewed as a feature of the genre of research which most often investigates problems in healthcare, rather than models of excellence. What are of interest here are the relativities: the fact that some concepts in some sub-periods are more positive than others. It is of interest to find that the more dominant concepts in the health services literature indicated by stage one of the analysis (patients, hospital, medical, clinical) tend to be the more positive concepts. Nursing, a concept that stages one and two suggested is less well connected to patient concepts, is also a less positive concept in these abstracts, when proportion of positive to negative sentiment is considered. This raises the question of whether the disassociation of nursing from patient care, observed in these abstracts in stages one and two, is somehow linked to the less positive nature of the construction of the nursing concept.

## Discussion

The most important and contested element of nursing identity for the wider public may be the patient-centredness of nursing, though this concept has had relatively less attention in the nursing identity literature focussing on the academicisation and specialisation of nursing, as well as identity formation in student learning. If nursing identity is a critical part of healthcare reform agendas, health service research is actively working to construct nursing identity in particular ways. The field of computational text analytics, designed for the surveillance of large qualitative datasets, offers new opportunities for nursing to scrutinise and regulate how its identity is constructed in large research and other databases.

As health services research has grown in importance as part of the translational research movement, a number of techniques of nursing identity construction can be observed in its corpus of abstracts, indicative of the larger corpus of publications. Nursing appears constructed within quality assurance or service implementation or workforce development concepts. It appears relatively disconnected from patient, family or community care concepts. The patient concept, which is central to health services research, appears to have been appropriated within biomedical interventionist, cost utility and research measurement discourses. At the same time, health service research appears to have also positioned nursing in ways disconnected from technical clinical disease and diagnostic concepts as well as more technical epidemiological concepts. These other apparent appropriations of nursing identity are also potentially significant. For those who value an idea of nursing identity as being about patient-centred caregiving, the apparent disconnection of nursing from patient, family or community care concepts potentially threatens its relevance to its client communities. From this perspective, the disconnect of nursing from technical clinical concepts as well as more technical epidemiological concepts potentially jeopardises nursing identity in two other critical ways. That is, the disconnect of nursing from clinical concepts could be seen as suggesting a repositioning of nurses as subordinate to clinical experts. The disconnection of nursing from technical epidemiological concepts could also arguably be seen as locating nursing away from critical 21^st^ century research techniques of power for shaping policy-making, described in Porter’s influential historical account of quantitative methodologies in policy-making: *Trust in Numbers: The Pursuit of Objectivity in Science and Public Life* [79].

The most ambiguous findings lie in the sentiment analysis. Given the well-established complexities and shortcomings of both human and computer-driven sentiment analyses [80], the sentiment analysis should be treated cautiously as an extension of stages one and two. Yet, in light of the findings of the earlier stages, it does raise important questions. Is the relative marginalisation of nursing in health services research, away from the central patient concept, as well as dominant clinical concepts, somehow caused by or leading to the use of the nursing concept in less positive ways? That is, put colloquially, is taking the care out of nursing linked to not caring about nursing?

Whatever the answer to this question, this study has offered indicative evidence of how the patient-centredness of nursing has fared in health service research captured by PubMed. It is precisely because health services research is a language space in which the nature of, and evidence for, healthcare is actively constructed that the general separation of the nursing concept from the patient concept is highly significant. The study could be seen as suggesting, in empirical quantifiable terms, a historic shift of nursing identity as direct caregiving. This shift can be seen in health services research represented in this study despite longstanding calls for nursing to be re-established as being about primary caregiving [81]. This study therefore suggests that health services evidence — itself a historical artefact that reflects cultural values and politicised language games — is working to create a quite different identity for nursing, at least in this database.

### Limitations

The fact that the study has been limited to the database PubMed — albeit a large and influential database — means its findings cannot be generalised to the entire discipline of health services, only health services research captured in PubMed. Accordingly, while the study allows one kind of “bird’s eye” view of over a quarter of a century of health services research represented by PubMed, it does not offer an opportunity to examine why or how nursing identity has come to be constructed in these ways within the health services literature, at least as it is contained in this database. Computational text analytics allows the analyst to quantify the content of a large qualitative dataset but it is less helpful in understanding how such constructs came about. Other quantitative and qualitative methods such as multivariate analysis [34] and critical discourse analysis [82,83], already used in nursing identity research [84], are needed to further explore the findings of this study. The contribution of countries and disciplines also are potentially fertile areas that lie outside the scope of the study. The value of the study therefore lies primarily in the way it offers the first scoping study of how health services research has positioned nursing identity in one particularly influential health sciences database.

## Conclusions

This study raises some important questions for the discipline of health services research, as well as nursing advocacy, research and education. It suggests that a key evidence-base for health services — PubMed — tends not to position nurses as patient-centred caregivers. In so doing, this study contributes to broad popular and scholarly debates about whether the nursing professions need to actively reappropriate constructs of patient care as more central to nursing identity. In such debates, as the introduction suggested, the importance of education and training, and further re-interpretation of the academicisation and professionalization of nursing identity, is already acknowledged. Less well-known is whether and how the evidence-base for health services itself has positioned nurses as patient-centred caregivers. For those who agree that patient-centredness should be a part of nursing identity in practice, this study suggests that there is a need for the development of health services research into both the nature of the caring construct in nursing identity and its expression in practice. One way this could happen is through more dedicated programs of health services research in nursing disciplines, in ways that better articulate patient-centredness. More fundamentally, the study raises questions about whether health services research cultures even value the politically popular idea of nurses as patient-centred caregivers, as this growing scholarly discipline enters the 21^st^ Century, and whether they should.
